# In Vitro Rescue of the Bile Acid Transport Function of ABCB11 Variants by CFTR Potentiators

**DOI:** 10.3390/ijms231810758

**Published:** 2022-09-15

**Authors:** Elodie Mareux, Martine Lapalus, Amel Ben Saad, Renaud Zelli, Mounia Lakli, Yosra Riahi, Marion Almes, Manon Banet, Isabelle Callebaut, Jean-Luc Decout, Thomas Falguières, Emmanuel Jacquemin, Emmanuel Gonzales

**Affiliations:** 1 Inserm UMR_S 1193, Physiopathogénèse et Traitement des Maladies du Foie, Université Paris-Saclay, FHU Hepatinov, 91400 Orsay, France; 2 Université Grenoble Alpes, CNRS, UMR CNRS 5063, DPM, 38000 Grenoble, France; 3 Assistance Publique-Hôpitaux de Paris, Pediatric Hepatology & Pediatric Liver Transplant Department, Reference Center for Rare Pediatric Liver Diseases, FILFOIE, ERN Rare-Liver, Faculté de Médecine Paris-Saclay, CHU Bicêtre, 94270 Le Kremlin-Bicêtre, France; 4 Muséum National d’Histoire Naturelle, UMR CNRS 7590, Institut de Minéralogie, de Physique des Matériaux et de Cosmochimie (IMPMC), Sorbonne Université, 75005 Paris, France

**Keywords:** BSEP, cholestasis, VX-770, potentiator, ABC transporter, pediatrics

## Abstract

ABCB11 is responsible for biliary bile acid secretion at the canalicular membrane of hepatocytes. Variations in the *ABCB11* gene cause a spectrum of rare liver diseases. The most severe form is progressive familial intrahepatic cholestasis type 2 (PFIC2). Current medical treatments have limited efficacy. Here, we report the in vitro study of Abcb11 missense variants identified in PFIC2 patients and their functional rescue using cystic fibrosis transmembrane conductance regulator potentiators. Three ABCB11 disease-causing variations identified in PFIC2 patients (i.e., A257V, T463I and G562D) were reproduced in a plasmid encoding an Abcb11-green fluorescent protein. After transfection, the expression and localization of the variants were studied in HepG2 cells. Taurocholate transport activity and the effect of potentiators were studied in Madin–Darby canine kidney (MDCK) clones coexpressing Abcb11 and the sodium taurocholate cotransporting polypeptide (Ntcp/*Slc10A1*). As predicted using three-dimensional structure analysis, the three variants were expressed at the canalicular membrane but showed a defective function. Ivacaftor, GLP1837, SBC040 and SBC219 potentiators increased the bile acid transport of A257V and T463I and to a lesser extent, of G562D Abcb11 missense variants. In addition, a synergic effect was observed when ivacaftor was combined with SBC040 or SBC219. Such potentiators could represent new pharmacological approaches for improving the condition of patients with ABCB11 deficiency due to missense variations affecting the function of the transporter.

## 1. Introduction

ABCB11, or the bile salt export pump (BSEP), is an adenosine tri-phosphate (ATP)-binding cassette (ABC) transporter localized at the apical (or canalicular) plasma membrane of hepatocytes. It allows normal bile flow through its essential function of biliary bile acid (BA) secretion [1]. ABCB11 belongs to the ABC transporter superfamily. These transporters are composed of two membrane-spanning domains (MSDs) that confer substrate specificity and provide the translocation paths for ligands and two nucleotide-binding domains (NBDs) involved in ATP binding and hydrolysis, thus providing energy for the active transport of BAs [2,3]. However, ABCB11 has asymmetric nucleotide-binding sites (NBS), with NBS-a being degenerated and expected to be catalytically inactive, with long-term ATP-binding at the NBD interface [4].

The dysfunction of ABCB11 leads to cholestatic liver diseases, the most severe form being progressive familial intrahepatic cholestasis type 2 (PFIC2), which is a rare autosomal recessive disease affecting patients harboring a homozygous or a compound heterozygous status for *ABCB11* variations early during childhood [1,3]. It is characterized by a decreased bile flow, BA accumulation in hepatocytes, ongoing hepatocellular damages and an increased risk of hepatocellular carcinoma. Clinical signs of cholestasis usually appear within the first months of life with jaundice and pruritus. Medical therapy with ursodeoxycholic acid and surgical therapy such as biliary diversion may provide some symptomatic relief. Nevertheless, none of these treatments is curative, and liver transplantation is required before adulthood for two thirds of PFIC2 patients [1,5]. This lack of effective treatment options highlights the need to identify new targeted pharmacotherapies as an alternative to liver transplantation for patients with severe forms of ABCB11-related diseases.

At present, almost 400 distinct variations of the *ABCB11* gene have been identified (https://gnomad.broadinstitute.org/; https://evs.gs.washington.edu/EVS/ and http://abcmutations.hegelab.org/, all accessed on 1 June 2022), including nonsense, missense and splicing variations, small insertions, deletions and duplications [5,6,7,8,9]. In a recent series reporting on 264 patients with ABCB11 variations, 208 (78%) carried at least one missense variation that can affect the traffic, function and/or the stability of the transporter [5]. For the latter two possibilities, a specific targeted pharmacotherapy could be proposed by using potentiators able to enhance the residual function of the transporter that is correctly localized to the bile canaliculi but is not, or less, functional. In vitro, we have recently shown the effectiveness of this strategy on Abcb11 missense variants: ivacaftor (VX-770, Kalydeco^®^), a clinically approved potentiating treatment for cystic fibrosis patients with some class III cystic fibrosis transmembrane conductance regulator (CFTR, *ABCC7*) variations, was able to potentiate the function of the Abcb11-T463I and Abcb11-A257V variants [10,11,12]. In the present study, we characterized another ABCB11 variant (G562D) and, in a repositioning strategy, we evaluated the effect of other CFTR potentiators (GLPG1837 and the small binders of CFTR SBC040 and SBC219) shown to rescue the functional defect of the G551D variant of CFTR [13,14]. Similar to the T463I and A257V variants, the G562D variant was correctly targeted to the canalicular membrane but displayed a BA transport function defect. In this context, the different tested CFTR potentiators were able to correct the functional defect of all three Abcb11 missense variants investigated in the present study.

## 2. Results

### 2.1. Patients

The A257V, T463I or G562D variations of ABCB11 were identified at a heterozygous status in young PFIC2 patients. The main characteristics of the patients are shown in Table 1.

### 2.2. Three-Dimensional Structure Analysis Predicts a Functional Defect of ABCB11 Variants

**Figure 1:**
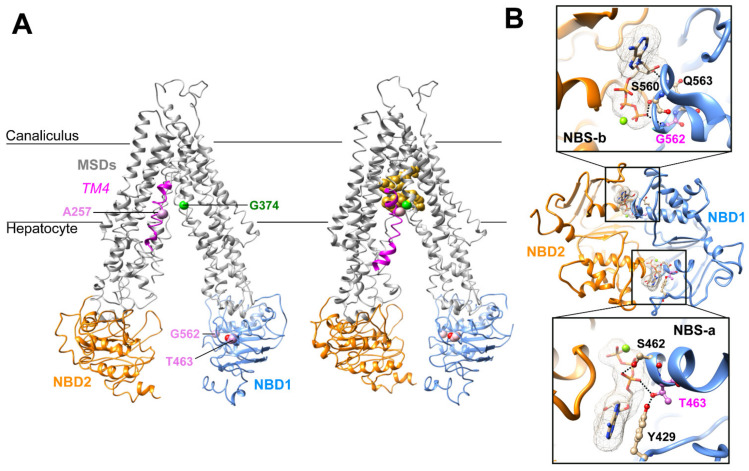
Three-dimensional structure analysis of human ABCB11. (**A**) Cryo-electron microscopy 3D structures of human ABCB11-wild type (wt) in an inward-open state, in apo (left) and taurocholate-bound (right) forms (PDB ID: 6LR0, 3.5 Å resolution [18]; 7DV5, 3.5 Å resolution [16], respectively). The taurocholate molecules are represented in gold. Ala257 (A257) belongs to the MSD1 TM4 (colored in magenta), whereas the Thr463 (T463) and Gly562 (G562) belong to the NBD1 (in blue). NBD2 is shown in orange. (**B**) Ribbon representation of the 3D structure model of ABCB11 NBD1/NBD2 assembly, built on the basis of the experimental 3D structure of human P-glycoprotein (ABCB1), in an outward-facing state (pdb 6C0V, 3.4 Å resolution [15]. The magnesium ions are represented as green spheres. Thr463 (pink) resides in the degenerated ATP-binding site “NBSa”, whereas Gly562 (pink) belongs to the consensus ATP-binding site “NBSb”. An alternate side chain rotamer to that present in the ABCB1 template was considered for Thr463, based on our previous model based on the MJ0796 3D structure, solved at higher resolution (1.9 Å) [10]. MSD, membrane-spanning domain; NBD, nucleotide-binding domain; TM, transmembrane helix.

The A257V and T463I variations and their potential defect were previously reported [10,11]. In brief, Ala257 resides in a flexible loop interrupting the transmembrane helix 4 (TM4) of ABCB11, as observed in the experimental three-dimensional (3D) structure of ABCB11 in an inward-facing conformation (Figure 1A). This region is predicted to refold in a continuous alpha helix in the outward-facing state, according to the comparison with the experimental 3D structure of the human P-glycoprotein (ABCB1) [15]. The flexibility of this region was further highlighted in the recent experimental 3D structure of ABCB11 in the presence of taurocholate [16], in which an induced fit was observed upon substrate binding, leading to a tight packing of TM4 at the level of Ala257 with TM6 Gly374 (Figure 1A).

Thr463 resides in the NBD1, inside the degenerated NBS-a and makes H-bonds with the hydroxyl group of A-loop Tyr429 (which stacks the ATP adenine) and, depending on the considered side chain rotamer, with the ATP α-phosphate oxygen atom (Figure 1A,B). The G562D variation is homologous to the gating-deficient variation G551D of CFTR [10]. Gly562 belongs to the NBD1 and is located within the NBS-b, and its backbone nitrogen atom makes an H-bond with ATP γ-phosphate oxygen (Figure 1A,B). In this position, there is not enough room to accommodate a larger side chain as the one of aspartate, leading to a steric hindrance when the Gly562 is mutated into an aspartate. The analysis of the 3D structures thus predicted that all three variations might directly or indirectly impact the ABCB11 transport function by disrupting ATP binding (T463I, G562D) or impairing ABCB11 local conformational change between the inward- and outward-facing states and upon substrate binding (A257V).

### 2.3. Abcb11 Variants Are Correctly Targeted to the Canalicular/Apical Membrane

**Figure 2:**
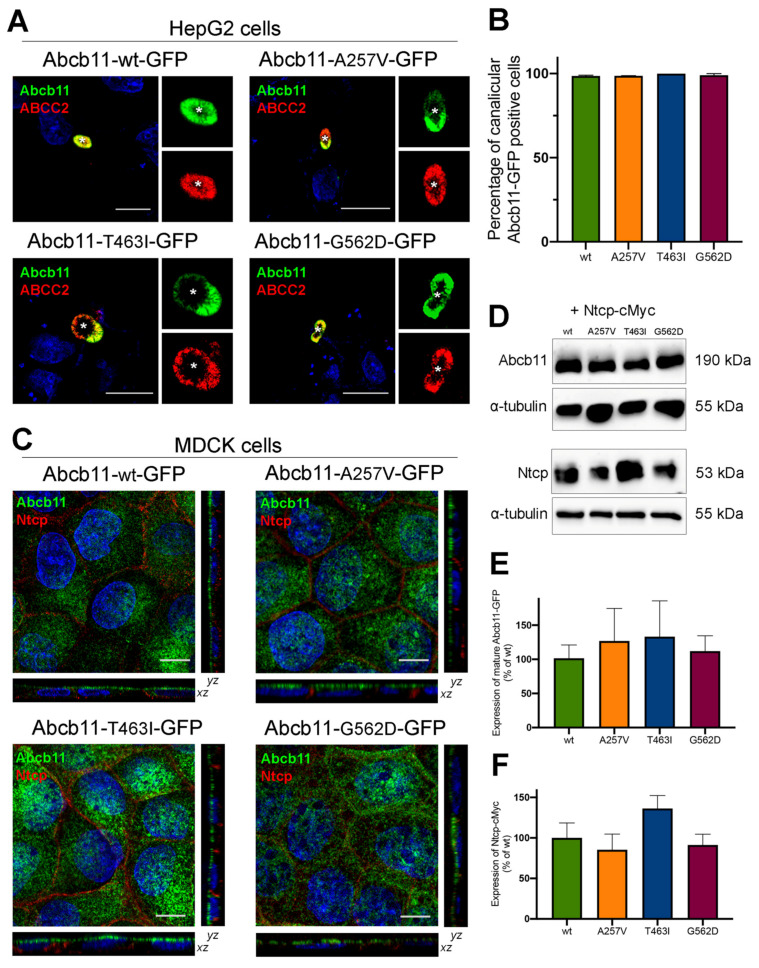
Abcb11 variants are expressed at the canalicular/apical membrane. (**A**) HepG2 cells were transiently transfected with Abcb11-GFP-encoding plasmids (wt and variants). Twenty-four hours later, cells were fixed and permeabilized. Immunolabelling of Abcb11-GFP (green) and endogenous ABCC2 (red) was visualized and analyzed using confocal microscopy. Nuclei shown in the merged images were labelled with Hoechst 33,342 (blue). This panel is representative of at least three independent experiments per condition. Dashed squares indicate magnification shown in the small right panels. Stars indicate canalicular structures. Bars: 10 µm. (**B**) Quantification of (**A**). Among Abcb11-GFP-positive cells forming canaliculi, the percentage of cells expressing Abcb11-GFP at the canalicular membrane was determined. Results are means (±SEM) of at least three independent experiments per condition. (**C**) MDCK clones stably expressing Abcb11-GFP (wt or variants) and Ntcp-cMyc were fixed and permeabilized. Immunolabelling of Abcb11-GFP (green) and Ntcp-cMyc (red) was analyzed using confocal microscopy. Nuclei were labelled with Hoechst 33,342 (blue). Bottom, center and right panels show x-z, x-y and y-z plane images, respectively. Bars: 10 µm. Each immunoblot is representative of at least three independent experiments per condition. (**D**) Cell lysates from MDCK clones stably expressing Abcb11-GFP (wt or variants) and Ntcp-cMyc were prepared and analyzed by immunoblotting using anti-GFP, anti-cMyc and anti-tubulin antibodies. This panel is representative of at least five independent experiments for each condition. (**E**,**F**) Densitometry analysis of Abcb11-GFP (**E**) and Ntcp-cMyc (**F**) from (**D**). Band intensities were separately quantified, and their relative amounts were calculated, normalized to α-tubulin levels and expressed as percentages of the Abcb11-wt-GFP condition. Means (± SEM) of at least three independent experiments per condition are shown. No statistical difference has been observed.

The canalicular expression of the A257V and T463I variants of Abcb11 were previously reported in Can 10 and/or HepG2 cells [10,11,17]. Twenty-four hours after transient expression of green fluorescent protein (GFP)-tagged Abcb11 in polarized HepG2 cells, we compared the expression of the variants to the wild type (wt). As with the wt protein and A257V variant, the T463I and G562D variants were exclusively expressed at the canalicular membrane of HepG2 cells and colocalized with endogenously expressed ABCC2, used as a canalicular marker of these polarized cells (Figure 2A, quantification in Figure 2B).

We then shifted to Madin–Darby canine kidney (MDCK) cells, a suitable model to measure BA vectorial transport [10,11]. In these polarized cells, stably expressed Abcb11-GFP (wt, A257V, T463I and G562D) was predominantly located along the apical membrane, and Ntcp-cMyc was exclusively located at the basolateral membrane (Figure 2C). On the immunoblot from MDCK cell lysates, Abcb11-GFP (wt, A257V, T463I and G562D) was present as a fully glycosylated protein with an apparent molecular weight of 190 kDa, and Ntcp-cMyc was detected at 53 kDa (Figure 2D). The absence of the detection of immature (not fully glycosylated) forms of the three variants of Abcb11 (Figure 2D) is in line with their canalicular expression. The quantification of these immunoblots indicated that expression levels of Abcb11-GFP and Ntcp-cMyc in MDCK clones were not statistically different (Figure 2E,F). Altogether, these results suggest that the A257V, T463I and G562D variations do not impair the intracellular trafficking of Abcb11.

### 2.4. The Three Variations of Abcb11 Do Not Impact the Stability of the Transporter

**Figure 3:**
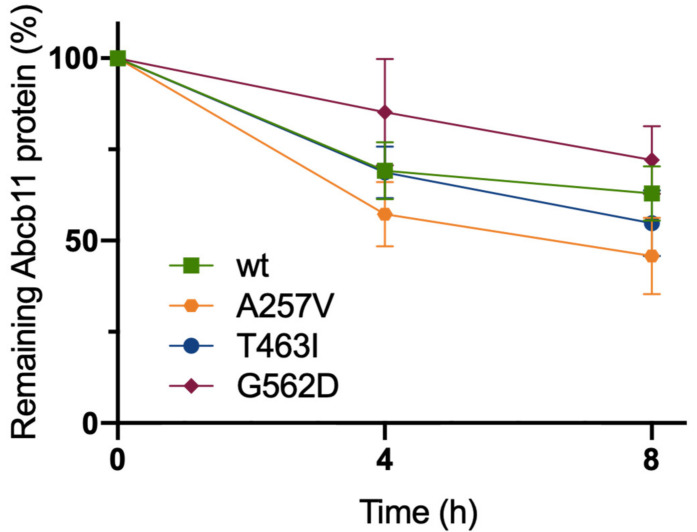
Abcb11 variants do not display significant stability defect. MDCK cells stably expressing Abcb11-GFP (wt or variants) were treated with cycloheximide (25 μg/mL) to inhibit protein synthesis. Then, expression of Abcb11-GFP (wt and variants) was analyzed using immunoblot at the indicated time points (Figure S1). Electrophoretic patterns of Abcb11-GFP were separately quantified at 0, 4 and 8 h after cycloheximide addition, and their relative amounts were calculated and normalized to α-tubulin. The amount of Abcb11-GFP at time 0 was considered as 100%. Remaining Abcb11-GFP after 4 and 8 h of treatment was expressed as a percentage of time point 0. Means (±SEM) of three independent experiments are shown.

To further characterize the molecular defect due to A257V, T463I and G562D variations, we assessed the stabilities of Abcb11 variants. The stabilities of the different forms of the transporter (wt and variants) were compared by analyzing the protein decay of Abcb11-GFP after the inhibition of protein synthesis with cycloheximide. At time point 0, Abcb11-GFP (wt and variants) appears as a fully glycosylated protein at 190 kDa (Figure S1). The quantification of these results showed that protein synthesis inhibition under cycloheximide treatment led to the progressive disappearance of Abcb11-wt-GFP (Figure 3). After 4 h, the wt showed a 31% decrease compared to the time point 0 and a reduction of 37% after 8 h (Figure 3). No significant difference was observed between the wt and the variants of Abcb11-GFP (Figure 3). These results suggest that the variants of Abcb11 investigated in the present study are as stable as the wt transporter.

### 2.5. CFTR Potentiators Rescue the Functional Defect Due to Abcb11 Variations

**Figure 4:**
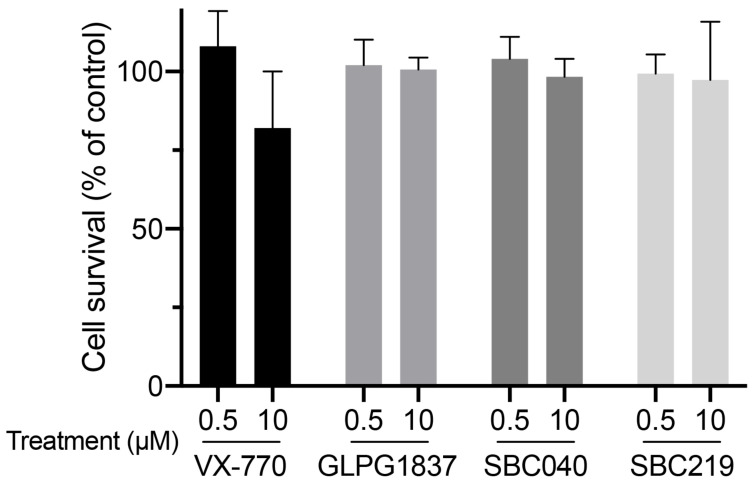
CFTR potentiators are not cytotoxic at 0.5 or 10 µM. MDCK cells were treated with 0.5 or 10 µM of the indicated potentiators for 2 h. Then, cytotoxicity was assessed using MTT assay and expressed as a percentage of the mean of the control (vehicle-treated cells). Means (±SEM) of at least three independent experiments (each performed in triplicate) per condition are shown.

Using a previously described approach with radiolabeled taurocholate (TC) [10], we measured Abcb11-GFP-mediated BA secretion of MDCK cells stably expressing different forms of the transporter (wt or variants) and after treatment with a vehicle (0.1% dimethylsulfoxide, DMSO) or CFTR potentiators (Table S1). We did not observe major cytotoxic effects of CFTR potentiators after 2 h of treatment at 0.5 or 10 µM in MDCK cells (Figure 4).

**Figure 5:**
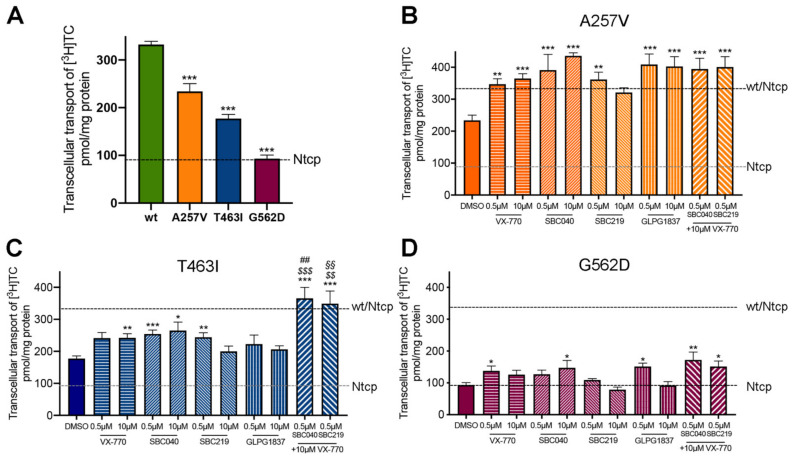
CFTR potentiators totally or partially rescue the functional defect of Abcb11 variants. (**A**–**D**) Vectorial transport of [^3^H]TC in MDCK cells stably expressing Abcb11-GFP (wt or variants) and Ntcp in the absence (**A**) or presence of the indicated concentrations of ivacaftor, GLPG1837, SBC040 or SBC219 in the A257V (**B**), T463I (**C**) or G562D (**D**) variants of Abcb11-GFP. The upper and lower dashed lines indicate [^3^H]TC transport measured in MDCK cells expressing both wt and Ntcp or Ntcp alone, respectively. Means (±SEM) of at least six independent experiments for each tested condition are shown. * *p* < 0.05; ** *p* < 0.01; *** *p* < 0.005 vs. non-treated Abcb11-wt-GFP expressing cells (panel A); ^$$^ *p* < 0.01; ^$$$^ *p* < 0.005 vs. VX 770-treated cells; ^##^ *p* < 0.01 vs. SBC040-treated cells; ^§§^ *p* < 0.01 vs. SBC219-treated cells.

As previously reported, A257V and T463I variants maintain a residual activity (70% and 53% of Abcb11-wt-GFP activity, respectively), while we observed a strongly reduced activity (28% of the wt transport activity) for the G562D variant in the absence of treatment (Figure 5A). All four CFTR potentiators allowed an increase from 1.5 to 1.9-fold of the BA transport activity of Abcb11-A257V-GFP, reaching the level of Abcb11-wt-GFP (Figure 5B). Except for GLPG1837, all CFTR potentiators significantly increased the BA transport activity of the T463I variant from 1.3 to 1.5-fold (Figure 5C). Only minor differences were observed between treatments at 0.5 or 10 µM. Lastly, BA transport activity of the G562D variant was significantly increased by treatment with ivacaftor at 0.5 µM (1.5-fold, *p* < 0.05), SBC040 at 10 µM (1.6-fold, *p* < 0.05) and GLPG1837 at 0.5 µM (1.6-fold, *p* < 0.05), to a lesser extent than the two other variants (Figure 5D). In addition, we tested whether the combination of treatments with ivacaftor and SBC potentiators might increase the BA transport activity of Abcb11 (Figure 5B–D, last two bars). A significant additive or synergetic effect between SBCs and VX-770 was observed for Abcb11-T463I-GFP (Figure 5C). Furthermore, a slight trend toward more BA transport activity was also observed for the G562D variant (Figure 5D). In MDCK cells expressing Ntcp and not Abcb11, we did not observe any significant alteration of Ntcp-mediated taurocholate transport after treatment with these drugs (our unpublished results). Altogether, these results indicate that each of the four CFTR potentiators tested, as well as combinations of SBCs with ivacaftor, are able to totally or partially rescue the functional defect of A257V, T463I and G562D Abcb11 variants.

## 3. Discussion

In this study, we further characterized three ABCB11 missense variants and evaluated the capability of four CFTR potentiators to correct their functional defect. The A257V, T463I and G562D missense variations were identified in heterozygous patients who developed PFIC2 during childhood. In previous studies, we have shown that the A257V and T463I variants are correctly targeted to the canalicular membrane but are not functional [10,11]. Although the G562D variant has a similar profile, its defective activity is stronger than those of the A257V and T463I variants. Therefore, its localization in the canonical NBS-b could be more crucial for ABCB11 function. Indeed, it has been shown that ABCB11 has asymmetric ATP-binding sites: NBS-b binds and hydrolyses ATP, while unable NBS-a is degenerated and is unable to hydrolyze ATP [4]. The latter point could allow the NBDs to be closer to allow multiple cycles of ATP hydrolysis [19]. Therefore, it is not surprising that a variation in a residue interacting with ATP in the NBS-b has a major effect on the transport function. Likewise, the homologous variation in CFTR (G551D) also shows an important functional defect [20,21]. Although 3D structure analysis indicates that Ala257 residue may play a critical role in ABCB11 local conformational changes between the inward- and outward-facing states and may thus indirectly impact its function, the A257V variant has the mildest phenotype of the three studied variants. Its localization in one of the MSDs instead of within the NBDs could have a minor impact on the transporter function, highlighting the importance of NBDs for the activity of the transporter as expected. Through analogy with ABCB4, a classification of the variations could be proposed: no protein expression (class I); intracellular retention (class II); impairment of BAs secretion activity (class III); defect of protein stability at the canalicular membrane (class IV); and no apparent defect (class V) [22]. Thus, the A257V, T463I and G562D variants would belong to class III variations.

Ivacaftor is FDA- and EMA-approved in selected cystic fibrosis patients harboring class III CFTR variations localized inside and outside ATP-binding sites [23]. We have previously shown the efficacy of ivacaftor at 10 µM to rescue the function of the T463I and A257V variants of Abcb11 [10,11]. Ivacaftor has been shown to rescue the channel activity of wt and mutated CFTR at subnanomolar concentrations in patch-clamp experiments [24], suggesting that we could use it at a lower concentration. In accordance with that, we show here that ivacaftor at 0.5 µM (as well as 10 µM) rescues the function of the A257V, T463I and G562D variants of Abcb11 but with different efficacies from one variant to another. These results show that ivacaftor may be clinically used to treat patients with class III *ABCB11* variations. The fact that a unique molecule can act on several transporters suggests common mechanisms of action among the ABC transporter superfamily. The binding of ivacaftor to the transporter could induce, for example, conformational changes that favor ATP binding. An ivacaftor binding site was first identified at the protein/lipid interface, involving TM4, 5 and 8 of CFTR [25]. The binding of ivacaftor to this site would facilitate the opening of the channel in an ATP-independent manner while prolonging its opening time in an ATP-dependent manner, resulting in increased Cl- ion secretion [12,26,27]. However, this site is absent from other ABC transporters, which could explain why ivacaftor potentiates the function of CFTR-wt but not the one of Abcb11-wt [10]. More recently, an ivacaftor binding site in the neighborhood of the CFTR intracellular loop 4 (ICL4) (between NBD1 and MSD2) has been proposed [28], but further investigations are required to determine whether this site is present in other ABC transporters. Another ivacaftor binding site was identified in the substrate-binding site of ABCB1 in the large central cavity that communicates with the cytoplasm and the lipid bilayer [29]. This site appears to be absent in CFTR, favoring the hypothesis that ivacaftor is a substrate of ABCB1 [30].

The development of new potentiating drugs is necessary to achieve higher clinical benefit for patients. Therefore, we were interested in promising new CFTR potentiators. GLPG1837 is currently evaluated in clinical studies (https://clinicaltrials.gov/ct2/show/NCT02707562; https://clinicaltrials.gov/ct2/show/NCT02690519, all accessed on 1 June 2022) [31]. It has been shown that this molecule enhanced the activity of class III CFTR variants to a higher extent than ivacaftor [32]. However, in our study, GLPG1837 showed similar efficacy to ivacaftor. GLPG1837 and ivacaftor have been shown to bind to the same CFTR site at the lipid/protein interface, which is absent in other ABC transporters [25]. Therefore, it is likely that the higher efficacy of GLP1837 compared to ivacaftor on CFTR is related to the presence of this binding site. It would be interesting to look for other GLPG1837 binding sites in CFTR and other ABC transporters and to determine whether these sites are the same as those for ivacaftor. More recently, the small binders of CFTR SBC040 and SBC219 have been developed to interact with an obvious target site on CFTR, i.e., filling the pocket known to be present in the altered ICL4:NBD1 interdomain interface in the F508del 3D structure [14]. Initially designed to be correctors, these compounds were found to have a potentiating activity on several class III CFTR variants sensitive to ivacaftor, whereas molecular dynamic simulations have proposed possible binding sites at the interface between NBDs [14]. In this study, we showed that these compounds were also able to potentiate the BA transport activity of A257V, T463I and G562D variants. In contrast to its homologous CFTR-G551D for which little or no potentiation was observed, the G562D variant of Abcb11 showed a positive response to SBC040. Moreover, SBC040 seems to restore the transport activity of the three Abcb11 variants to a greater extent than SBC219. Furthermore, we did not observe a stronger effect of SBC219 than ivacaftor, in opposition to what has been shown for CFTR potentiation [14]. The level of transport function after Abcb11-G562D variant potentiation remains lower than the one of the residual activity of T463I, suggesting that the potentiation of G562D may not be clinically significant. Finally, a potentiation of the function of the wt protein was observed in CFTR [14] but not for Abcb11 (our unpublished results). Similarities were also observed such as the synergistic effect of the combination of ivacaftor and SBCs, suggesting different binding sites than those for ivacaftor. Hence, SBCs may modulate the CFTR channel and ABCB11 transporter using different mechanisms. In the framework of forthcoming studies, it, therefore, would be interesting to study the potential binding sites of these compounds in ABCB11 and investigate the effect of these potentiators on the ATPase activity of ABCB11 (wt and variants).

The present in vitro study enabled the further characterization of three ABCB11 variants and the identification of new potentiating drugs (SBC040, SBC219 and GLPG1837) able to correct the functional defects caused by ABCB11 variations. This constitutes a proof of concept for repurposing CFTR potentiators, alone or in combination, as a new therapeutic option for selected patients with ABCB11 deficiency caused by class III variations, i.e., affecting the function of the transporter. Considering that there is an unmet medical need for patients with BSEP/ABCB11 deficiency, this could represent a significant step forward for the care of such selected patients.

## 4. Materials and Methods

### 4.1. Patients

General information regarding the patients and *ABCB11* gene analysis have been previously reported and are summarized in Table 1 [6,10,17].

### 4.2. Three-Dimensional Analysis

The 3D structures of ABCB11 in inward-facing conformation were available in an apo form (pdb 6LR0) [18] and in complex with taurocholate (pdb 7DV5) [16]. The model of the 3D structure of the ABCB11 NBD1/NBD2 assembly was built with Modeller v9.15 (University of California, San Francisco, CA, USA) [33] using, as a template, the experimental structure of ABCB1, determined in the outward facing conformation using cryo-electron microscopy at 3.4 Å resolution and with the NBD1/NBD2 dimer in complex with ATP (pdb 6C0V) [15] (65% sequence identity). 3D structure coordinates were manipulated and visualized using Chimera (https://www.cgl.ucsf.edu/chimera/, accessed on 1 April 2022). The membrane position was predicted using the PPM 3.0 web server (https://opm.phar.umich.edu/ppm_server3, accessed on 1 April 2022).

### 4.3. DNA Constructs and Mutagenesis

The C-terminus GFP plasmids encoding wt and missense variants (A257V and T463I) of Abcb11 have been described, as well as the C-terminus cMyc plasmid encoding Ntcp [10,17]. The primers used for G562D mutagenesis were: 5′-AGGCCAGATGAGTGGT**GAT**CAGAAGCAAAGAGTAG-3′ (forward) and 5′-CTACTCTTTGCTTCTG**ATC**ACCACTCATCTGGCCT-3′ (reverse).

### 4.4. Cell Culture, Transfection, Lentiviral Infection and Immunoanalyses

GFP-tagged Abcb11-encoding vectors (wt, A257V, T463I or G562D) were transiently transfected in human hepatocellular carcinoma HepG2 cells as published [34]. These cells form pseudo-bile canaliculi in culture and allow localization studies [35]. MDCK cells were stably transfected as described [10]. MDCK clones with the highest Abcb11-GFP (wt, A257V, T463I or G562D) expression and parental MDCK cells were infected with Ntcp- encoding lentiviral particles as described [10]. Immunofluorescence and immunoblotting analyses were performed as described [10] using the following primary antibodies: rat monoclonal anti-cMyc (Clone JAC6; GeneTex, Irvine, CA, USA), rabbit polyclonal anti-GFP (ab290; Abcam, Cambridge, UK), mouse monoclonal anti-ABCC2 (clone M2I-4; Enzo Life Sciences, Villeurbanne, France), mouse monoclonal anti-GFP (clone 7.1 and 13.1; Roche Diagnostics, Mannheim, DE, USA), anti-cMyc (Clone 9E10; BD Biosciences Pharmingen, San Diego, CA, USA) and anti-α-tubulin (clone 1E4C11; ProteinTech, Manchester, UK). Peroxydase- and fluorochrome-conjugated secondary antibodies were from GE Healthcare (Chicago, IL, USA) and Molecular Probes/Thermo Fisher Scientific (Illkirch, France), respectively.

### 4.5. Chemicals and Cell Treatments

Ivacaftor and GLPG1837 were from Selleckchem (S1144 and S8698, respectively, Munich, DE, USA). SBC040 and SBC219 were synthesized by J.L. Decout (University Grenoble Alpes, France). All compounds were solubilized in DMSO as 1000 X concentrated stock solutions to treat cells with 0.5 or 10 µM final concentration, using DMSO as a control vehicle at the same dilution (0.1% DMSO for all conditions). After two hours of treatment with these drugs, cells were used for cytotoxicity or TC transport assays (see below).

### 4.6. Analysis of Abcb11 Protein Stability

MDCK cells stably expressing Abcb11-GFP (wt or variants) were treated with 25 µg/mL cycloheximide to inhibit further protein synthesis. Following incubation for 0, 2, 4, 6 or 8 h, cells were harvested, lysed and submitted to SDS-PAGE and immunoblot analysis. Band densities were quantified using ImageJ software v1.41 (National Institutes of Health, Bethesda, MD, USA) and normalized by comparison with time point 0.

### 4.7. Cytotoxicity Assays

Cytotoxicity of CFTR potentiators was assessed by the conversion of MTT (3-[4,5-dimethylthiazol-2-yl]-2,5 diphenyl tetrazolium bromide; Sigma-Aldrich/Merck, Saint-Quentin-Fallavier, France) into formazan crystals using living cells, as described [36]. Briefly, subconfluent MDCK cells were plated into 96-well plates in triplicate for each tested condition, including controls (no cells and 0.1% DMSO). Sixteen hours after cell seeding and 2 h after drug treatment, MTT (125 µg/mL, final concentration) was added in each well, and cells were reincubated at 37 °C for 2 h. Then, culture medium was gently washed out, and cells were lysed in 100 µL DMSO, and the absorbance at 540 nm was measured using a Wallac Victor^3^ multilabel plate reader (Perkin Elmer, Massy, France). Cell survival was calculated for each triplicate, and, after background subtraction, means were expressed as percentages of the mean of cells treated with the vehicle only.

### 4.8. Taurocholate Transport Assays

Measurement of Abcb11-GFP-mediated TC secretion was performed in polarized MDCK cells grown on membrane inserts, as described [10]. In brief, culture medium was replaced by prewarmed transport buffer in apical and basal compartments, in the absence or presence of 0.5 or 10 µM of ivacaftor, GLPG1837, SBC040 or SBC219. [^3^H]TC (Perkin Elmer) was added in the basal compartment (in the wells). After two hours, the apical buffer (in the membrane inserts) was collected, and transcellular transport of [^3^H]TC was calculated from the radioactivity present in the apical buffer. Transport data were normalized to protein amounts determined by bicinchoninic acid assay (QuantiPro™ BCA Assay Kit, Sigma-Aldrich/Merck).

### 4.9. Statistical Analyses

Data are expressed as means ± standard error of the mean (SEM). Graphics and statistical analyses were performed using one-way ANOVA using Prism version 7.00 (GraphPad software, La Jolla, CA, USA). A *p* value < 0.05 was considered significant.

## Figures and Tables

**Table 1:** Features of PFIC2 patients with ABCB11 variations.

Patient (No./Sex)	First Allele	Domain	Second Allele	Domain	Biliary BA (N < 10 µM)	IHC ABCB11	Reference
1/F	c.770C > T p.A257V	TM4	c.2944G > A p.G982R	TM11	na	na	[17]
2/F	c.1388C > T p.T463I	NBD1 NBS-a	c.3169C > T p.R1057X	Linker TM6-NBD2	1 mM	Faint	[10]
3/na	c.1685G > A p.G562D	NBD1 NBS-b	c.1445A_G p.D482G	NBD1	na	Normal	[6]

Nucleotide variant corresponds to the cDNA of the NM_003742.4 (ABCB11, transcript variant A, mRNA). Abbreviations: BA, bile acids; F, female; na, not available; IHC, immunohistochemistry; NBD, nucleotide-binding domain; NBS, nucleotide-binding site; TM, transmembrane helix.

## Data Availability

The datasets used and/or analyzed during the current study are available from the corresponding author on reasonable request.

## Supplementary Materials

The following supporting information can be downloaded at: https://www.mdpi.com/article/10.3390/ijms231810758/s1.

## Abbreviations

ABC	ATP-binding cassette
BA	Bile acid
BSEP	Bile salt export pump
CFTR	Cystic fibrosis transmembrane conductance regulator
DMSO	Dimethylsulfoxide
MDCK	Madin–Darby canine kidney
MSD	Membrane-spanning domain
NBD	Nucleotide-binding domain
NBS	Nucleotide-binding site
NTCP	Na^+^-taurocholate cotransporting polypeptide
PFIC	Progressive familial intrahepatic cholestasis
SBC	Small binder of CFTR
TC	Taurocholate
TM	Transmembrane helix
WT	Wild type
