# Supplementary material for: In Vitro Rescue of the Bile Acid Transport Function of ABCB11 Variants by CFTR Potentiators
Source: Int J Mol Sci. 2022 Sep 15;23(18):10758. doi: 10.3390/ijms231810758 (PMC9502978; doi:10.3390/ijms231810758)
Supplement: Supplementary file 1 [file ijms-23-10758-s001.zip › ijms-1887227-supplementary.pdf]

SUPPLEMENTARY INFORMATION

In vitro Rescue of the Bile Acid Transport Function of ABCB11 Variants by CFTR Potentiators

Elodie Mareux[1], Martine Lapalus[1], Amel Ben Saad[1], Renaud Zelli[2], Mounia Lakli[1], Yosra Riahi[1], Marion Almes[3], Manon Banet[1], Isabelle Callebaut[4], Jean-Luc Decout[2], Thomas Falguières[1,†], Emmanuel Jacquemin[1,3,†], Emmanuel Gonzales[1,3,*,†]

[1] Inserm UMR_S1193, Physiopathogénèse et traitement des maladies du foie, Université Paris-Saclay, FHU Hepatinov, 91400 Orsay, France.

[2] Université Grenoble Alpes, CNRS, UMR CNRS 5063, DPM, 38000 Grenoble, France.

[†] These authors share last co-authorship.

[3] Assistance Publique - Hôpitaux de Paris, Pediatric Hepatology & Pediatric Liver Transplant Department, Reference Center for Rare Pediatric Liver Diseases, FILFOIE, ERN Rare-Liver, Faculté de Médecine Paris-Saclay, CHU Bicêtre, 94270 Le Kremlin-Bicêtre, France.

[4] Muséum National d'Histoire Naturelle, UMR CNRS 7590, Institut de Minéralogie, de Physique des Matériaux et de Cosmochimie (IMPMC), Sorbonne Université, 75005 Paris, France.

*** Corresponding author**: Pr Emmanuel Gonzales, M.D., PhD., Service d'Hépatologie et de transplantation hépatique pédiatriques, Hôpital Bicêtre, 78 rue du Général Leclerc, 94275 Le Kremlin-Bicêtre cedex, France. Tel: 33-1-45-21-37-88. E-mail: emmanuel.gonzales@aphp.fr

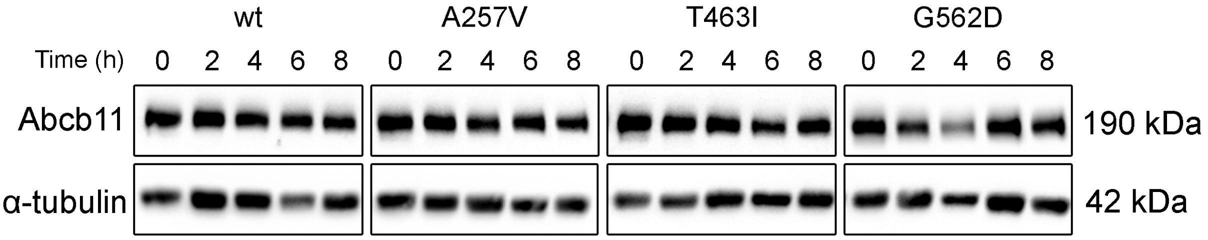

Figure S1: Stability of the Abcb11 variants. MDCK cells stably expressing Abcb11-GFP (wt or variants) were treated with cycloheximide (25 µg/mL) to inhibit protein synthesis. Then, expression of Abcb11 (wt and variants) and α-tubulin was analysed by immunoblot using anti-GFP and anti-tubulin antibodies at the indicated time points.

Table S1: Chemical structures of CFTR potentiators tested in this study.

Compound	IUPAC Name	Chemical structure	PubChem CID	MW (Da)	References
Ivacaftor (VX-770)	N-(2,4-ditert-butyl-5-hydroxyphenyl)-4-oxo-1H-quinoline-3-carboxamide	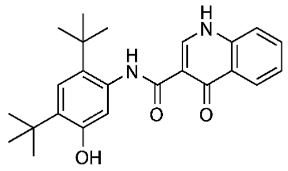	16220172	392.49	[1]
GLPG1837 (ABBV-974)	N-(3-carbamoyl-5,5,7,7-tetramethyl-4H-thieno[2,3-c]pyran-2-yl)-1H-pyrazole-5-carboxamide	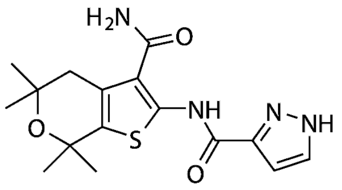	117857370	348.42	[2]
SBC040	4-(6-Amino-2-chloro-9H-purin-9-yl)benzamide	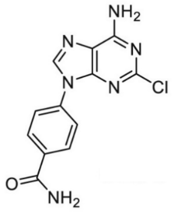	–	403.7	[3]
SBC219	4-(6-Amino-2-((3-phenylpropyl)amino)-9H-purin-9-yl)benzamide	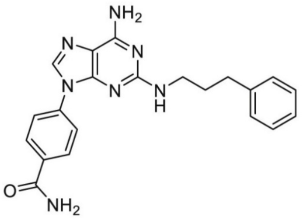	–	387.4	[3]

Supplementary References

1. Van Goor, F.; Hadida, S.; Grootenhuis, P. D.; Burton, B.; Cao, D.; Neuberger, T.; Turnbull, A.; Singh, A.; Joubran, J.; Hazlewood, A., et al. Rescue of CF airway epithelial cell function in vitro by a CFTR potentiator, VX-770. *Proc Natl Acad Sci U S A* **2009,** 106, 18825-30.
2. Yeh, H. I.; Sohma, Y.; Conrath, K.; Hwang, T. C. A common mechanism for CFTR potentiators. *J Gen Physiol* **2017,** 149, 1105-1118.
3. Froux, L.; Elbahnsi, A.; Boucherle, B.; Billet, A.; Baatallah, N.; Hoffmann, B.; Alliot, J.; Zelli, R.; Zeinyeh, W.; Haudecoeur, R., et al. Targeting different binding sites in the CFTR structures allows to synergistically potentiate channel activity. *Eur J Med Chem* **2020,** 190, 112116.
